# Supplementary material for: Association of Twelve Candidate Gene Polymorphisms with the Intramuscular Fat Content and Average Backfat Thickness of Chinese Suhuai Pigs
Source: Animals (Basel). 2019 Oct 23;9(11):858. doi: 10.3390/ani9110858 (PMC6912197; doi:10.3390/ani9110858)
Supplement: Supplementary file 1 [file animals-09-00858-s001.zip › Supplementary Materials.docx]

**Table S1.** Primer sequences and PCR products of 13 SNPs in 12 previously reported candidate genes for intramuscular fat content (IFC) in this study.

| **Gene (SNP)** | **Primer Sequence (5’→3’)** | **PCR Product (bp)** |
| --- | --- | --- |
| *FABP3* (rs1110770079) | F:ATTGCTTCGGTGTGTTTGAG | 693 |
|  | R:TCAGGAATGGGAGTTATTGG |  |
| *LIPE* (rs328830166) | F:CCTAGCTGCTCTCACCCAGCTC | 149 |
|  | R:AGCAGCCCTTGTGTAGAGTGACATAC |  |
| *IGF1* (rs341412920) | F:GTGGCACTGACTGCAGGAGAAA | 306 |
|  | R:TCTCCAGCCTCCTCAGATCACA |  |
| *IGF1* (rs322131043) | F:AATTGCCTGGTGAGGCATAAGA | 306 |
|  | R:GGGGAATGCCCATCTTTTGAA |  |
| *IGF2* (g.3072G>A) | F:AGGTGCCGTGGAGCTGGG | 144 |
|  | R:GGCGGCCTAGTTGCAGTAGTTC |  |
| *LEP* (rs45431504) | F:TCCTCTTGCCTGCTGGAATCTC | 209 |
|  | R:AATGCCTTCCCTGCAATGTTGT |  |
| *LEPR* (rs45435518) | F:GCTTGGCAGAGCCAACATCTCT | 165 |
|  | R:GTTGGTAGATCGCCAGGGTCTG |  |
| *MC4R* (rs81219178) | F:TTCTTCACCATGCTGGCTCTCA | 319 |
|  | R:GGCTCCGGAGTGCATAAATCAG |  |
| *PHKG1* (rs697732005) | F:GTGGTGGAGGAAGTGCGT | 140 |
|  | R:GTTCTGCTGCTGCCCCTT |  |
| *RETN* (rs327132149) | F:GGGAGGGGTAGCTTGGTCTCAG | 237 |
|  | R:GGCAGGGACTTGTGGTCCTAGA |  |
| *RYR1* (rs344435545) | F:TGTTCCCTGTGTGTGTGCAATG | 199 |
|  | R:TTCACCGGAGTGGAGTCTCTGA |  |
| *SCD* (rs80912566) | F:GCAGCGAATAAAAGGGGTCAGA | 318 |
|  | R:GGGGGCCATTACTTGGAAACTC |  |
| *UBE3C* (rs81329544) | F:AACCTGTCCTGTTGCTCTCA | 295 |
|  | R:CAGGAGAGCCAGCTAAACT |  |

*FABP3*: fatty acid binding protein 3; *LIPE*: lipase E, hormone sensitive type; *IGF1*: insulin like growth factor 1; *IGF2*: insulin like growth factor 2; *LEP*: leptin; *LEPR*: leptin receptor; *MC4R*: melanocortin 4 receptor; *PHKG1*: phosphorylase kinase catalytic subunit gamma 1; *RETN*: resistin; *RYR1*: ryanodine receptor 1; *SCD*: stearoyl-CoA desaturase; *UBE3C*: ubiquitin protein ligase E3C.
