# Supplementary figures and images for: Association of Twelve Candidate Gene Polymorphisms with the Intramuscular Fat Content and Average Backfat Thickness of Chinese Suhuai Pigs
Source: Animals (Basel). 2019 Oct 23;9(11):858. doi: 10.3390/ani9110858 (PMC6912197; doi:10.3390/ani9110858)

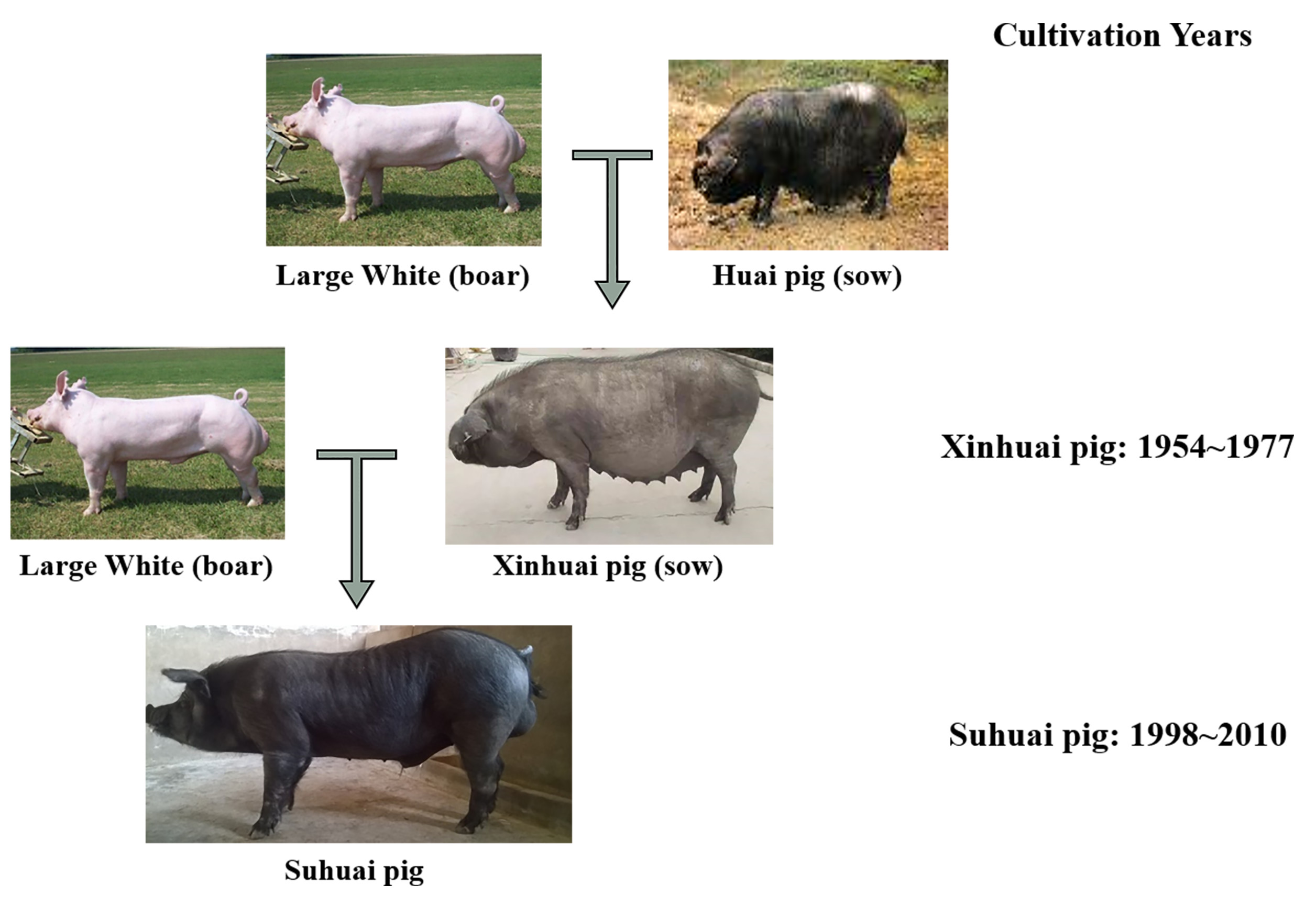

Supplement: Supplementary file 1 [file animals-09-00858-s001.zip › Figure 1. The cultivation process of Suhuai pig.jpg]
